# Metabolic Cost of a Nutritional Symbiont Manifests in Delayed Reproduction in a Grain Pest Beetle

**DOI:** 10.3390/insects11100717

**Published:** 2020-10-20

**Authors:** Tobias Engl, Thorsten H. P. Schmidt, Sthandiwe Nomthandazo Kanyile, Dagmar Klebsch

**Affiliations:** 1 Evolutionary Ecology, Institute of Organismic and Molecular Evolution, Johannes Gutenberg-University, 55128 Mainz, Germany; gts.schmidt@yahoo.de (T.H.P.S.); skanyile@uni-mainz.de (S.N.K.); klebsch@uni-mainz.de (D.K.); 2 Research Group Insect Symbiosis, Max-Planck-Institute for Chemical Ecology, 07745 Jena, Germany

**Keywords:** bacteroidetes, cuticle synthesis, metabolic and fitness cost, Oryzaephilus surinamensis, sawtoothed grain beetle, symbiosis

## Abstract

**Simple Summary:**

Animals engage in various symbioses. However, these interactions are not always beneficial for the host; they can also incur costs under certain circumstances. The bacterial symbiont supports, on the one hand, the cuticle formation of the sawtoothed grain beetle *Oryzaephilus surinamensis,* which is extremely beneficial under dry conditions as a thicker and more melanized cuticle prevents desiccation of the insect. On the other hand, under higher humidity, the benefit is strongly reduced. In this study, we investigated whether harboring a symbiont can also be a disadvantage. Therefore, we first measured the number of symbionts throughout the beetles’ life and found a strong increase during the end of metamorphosis, just before beetles reach adulthood. Afterwards, males lose the symbionts again, whereas females retain a stable number. A comparison of beetles with and without symbionts revealed no differences in many life history traits. Larval development took the same time and there was also no difference in adult mortality or lifespan or the number of offspring of females. However, females with symbionts started to reproduce significantly later by one to two weeks, meaning they have a disadvantage in comparison to females without symbionts. Thus, harboring a symbiont is beneficial or costly in a context-dependent manner.

**Abstract:**

Animals engage in a plethora of mutualistic interactions with microorganisms that can confer various benefits to their host but can also incur context-dependent costs. The sawtoothed grain beetle *Oryzaephilus surinamensis* harbors nutritional, intracellular Bacteroidetes bacteria that supplement precursors for the cuticle synthesis and thereby enhance desiccation resistance of its host. Experimental elimination of the symbiont impairs cuticle formation and reduces fitness under desiccation stress but does not disrupt the host’s life cycle. For this study, we first demonstrated that symbiont populations showed the strongest growth at the end of metamorphosis and then declined continuously in males, but not in females. The symbiont loss neither impacted the development time until adulthood nor adult mortality or lifespan. Furthermore, lifetime reproduction was not influenced by the symbiont presence. However, symbiotic females started to reproduce almost two weeks later than aposymbiotic ones. Thus, symbiont presence incurs a metabolic and context-dependent fitness cost to females, probably due to a nutrient allocation trade-off between symbiont growth and sexual maturation. The *O. surinamensis* symbiosis thereby represents an experimentally amenable system to study eco-evolutionary dynamics under variable selection pressures.

## 1. Introduction

Symbioses play a major role in the ecology and evolution of organisms [1,2]. Microbial symbionts can provide various benefits to their host, from nutrient supplementation and support in food digestion and detoxification to defense against natural enemies and adaptation to abiotic environmental conditions [3,4,5,6,7,8,9,10,11,12,13,14]. However, engaging in a symbiotic association can also be costly. While costs can manifest on different levels for an organism, they ultimately reduce the reproductive success in comparison to individuals that do not engage in this symbiotic association. Whether a certain symbiotic association is beneficial or costly for the host is often context dependent, which can thus result in fluctuating symbiont infection frequencies, titers, or even symbiont loss and replacement [9,15,16,17,18].

Physiological costs usually result from trade-offs. Metabolic costs arise through the limitation of nutrients and an allocation trade-off, e.g., between investment into reproduction and somatic maintenance, but also investment into immune defenses [19,20]. Insects harboring symbionts must additionally allocate nutrients to their symbionts. Nutrient investment is required for the biosynthesis of a beneficial metabolite, as long as it is not a so-called by-product, for which the producing partner has actually no further use [21]. In addition, the host also has to invest nutrients into the growth and maintenance of their symbiont pool that might determine the amount of any benefit they can get in return—often delayed by a considerable time. These nutrients are of course not available to the host for reproduction and any somatic processes—at least not for a given point in an organism’s life. Some hosts compensate this investment by degrading symbionts and reabsorbing nutrients in case their support is only needed for a certain life stage [22].

Ecological costs can arise from a limitation of the host in interactions with its biotic and abiotic environment that result from the association with its symbiont. The nutritional endosymbionts of, for example, whiteflies, aphids, and beetles are often more susceptible to elevated environmental temperatures than their host, which limits their ecological habitat [23,24,25,26,27,28]. Many symbionts also actively modulate or limit mating of their hosts [11,29,30]. Evolutionary costs, on the other hand, can arise from the co-adaptation of intimate associations over long time spans when metabolic versatility is lost, which happens often during genome degradation or streamlining and results ultimately in a metabolic dependency [31,32,33]. However, not only many endosymbiont bacteria suffer irreversibly from genome degeneration through fragmentation or gene loss, but pea aphids are also thought to have lost parts of their innate immune system in response to symbiotic interaction with defensive symbionts [34,35].

The potential and realized costs of symbiotic associations are mostly investigated in facultative defensive symbioses. Several publications highlight the costs that defensive symbionts represent for their hosts in the absence of natural enemies, due to metabolic demands that ultimately cause a fitness burden, e.g., through reduced fecundity or viability in comparison to non-infected individuals [36,37]. Nutritional symbioses in which the microbial partner supplies its host with nutrients that are limited or absent in its diet are less well investigated. Most nutritional symbioses are obligate for one or both partners, meaning that a partner cannot survive or reproduce on its own. Experimental studies thus depend usually on sophisticated artificial feeding systems that are often hard to establish [38,39,40]. Few model systems have so far been established in which microbial symbionts provide nutrients, but are not obligate for their host, either in laboratory conditions or, in some cases, where natural aposymbiotic host populations have been reported [23,24,41]. We recently demonstrated that a grain pest beetle, the sawtoothed grain beetle *Oryzaephilus surinamensis*, harbors Bacteroidetes symbionts that support their host by hardening the cuticle [42,43,44]. *O. surinamensis* is able to survive and reproduce in a quite broad range of environmental humidity conditions (at least from 12% to 90%) [45,46], partially supported by this mutualistic association—symbiotic beetles exhibited a humidity-dependent benefit in terms of lower larval mortality and stronger population growth compared to aposymbiotic beetles [44]. While the beetles could still survive and reproduce without their symbionts under low humidity (30% RH), they suffered a 50–70% fitness reduction. However, under high humidity (60% RH), the fitness benefit of carrying a symbiont population was considerably reduced (~25%) regarding both parameters [44].

For this study, we characterized the course of symbiont infection in different life stages of *O. surinamensis* and tested the impact of the symbiosis on several life history parameters, including development and mortality of larvae and adults as well as lifetime reproduction of females at reduced environmental stress. We specifically chose the lower stress, higher humidity of 60%, from our last study, as this represents an intermediate state of the humidity range under which *O. surinamensis* can thrive and as any costs of harboring symbionts and beneficial effects might be balanced and be both observable. Having already demonstrated benefits under higher abiotic stress, meaning low environmental humidity, we are able to evaluate factors selecting for or against the maintenance of the symbiont infection. We specifically demonstrate that symbiont infection does still confer minor benefits during juvenile development at high environmental humidity, but constitutes also metabolic costs in adult females, as the onset of reproduction is significantly delayed in symbiotic females in comparison to aposymbiotic ones.

## 2. Materials and Methods

### 2.1. General Insect Rearing and Symbiont Elimination

*O. surinamensis* beetles originated either from a long-term laboratory culture provided in 2014 by the Julius-Kühn-Institute/Federal Institute for Cultivated Plants (Berlin, Germany, subsequently termed ‘JKI’) or from natural populations caught 2 years before experiments in a university kitchen in Mannheim (Germany, termed ‘Mannheim’) and 1 year before experiments in Mainz (Germany, termed ‘Mainz’). Symbiotic stock populations were continuously maintained in our laboratory. Aposymbiotic cultures were first established in 2015 by rearing 150 beetles from the JKI stock for 3 months on a tetracycline-containing diet and transferring their offspring back to a standard oat flake diet and maintained since then (for details, see [44]). This procedure was replicated in 2018 and 2019 to create technical aposymbiotic replicates from the JKI stock cultures as well as biological replicates with additional strains that were not inbred for generations under laboratory conditions, resulting in total in the following experimental cultures: JKI1, JKI2, JKI3, Mannheim, and Mainz. A control treatment undergoing identical population bottle necks and food availability was established at the same time for each of the replicates [44]. The presence and absence of symbionts in the different populations were confirmed just before experiments started by quantitative PCR (see below). Beetle stock and experimental cultures were kept in 1.8 L plastic containers, filled with 50g oat flakes, at 28 °C and relative humidity between 40% and 60% in the dark.

### 2.2. Symbiont Influence on Development

Symbiont titer throughout development in symbiotic beetles as well as developmental time and survival of symbiotic and aposymbiotic beetles until adulthood were monitored by collecting females directly after maturation from JKI-1 experimental cultures and placing them individually in 12-well plates that were controlled daily for new eggs.

Symbiont titer was quantified throughout development in individuals of defined age. Therefore, symbiotic eggs were collected, separated, and incubated with daily observation to obtain individuals of defined life stages. Molting was scored by the presence of empty exuviae that were removed after each molting. Ten individuals of the following life stages were collected at day 1 of each stage, if not described otherwise: eggs, first, second, third, fourth, and fifth larval instar, pupae at days 1 and 5, females and males at the age of one week, one month, and three months after imagines hatched. Animals were kept under previously mentioned conditions except for a 1 h observation window each day. Food was provided ad libitum by coating the bottom of each well beforehand with a slurry of ground oat flakes (Bio-Hafergold, Holo, Germany) and distilled water that was dried overnight at 60 °C.

In a second separate experiment of identical initial setup, developmental time to reach different life stages was monitored, again using beetles from the JKI-1 experimental cultures. All eggs were transferred to individual wells of 24-well plates and again monitored daily for survival and development of the juvenile stages. Molting was scored by the presence of empty exuviae that were removed after each molting. Then, 24-well plates were coated with Fluon (a 60% polytetrafluoroethylene dispersion in water, Sigma-Aldrich, St. Louis, MI, USA) to prevent females from escaping and animals in both types of multi-well plates were provided with food ad libitum as in the first experiment. Animals were kept under previously mentioned conditions except for a 1h observation window each day.

Symbiont titer was measured by quantitative PCR (established in [44]). DNA was extracted using an Epicentre MasterPure Complete DNA and RNA Purification Kit (Illumina Inc., Madison, WI, USA) and redissolved in 20 µL low TE buffer (1 mM Tris, 0.1 mM EDTA). Quantitative PCRs were carried out in 25 μL reactions using the Qiagen QuantiTect-SYBR-Green-PCR mix (Qiagen, Venlo, The Netherlands), including 0.5 μM of each primer and 1μL template DNA using symbiont specific primer ‘OsurSym_fwd2′ and ‘mod.CFB563_rev’ [44]. Amplification followed a two-step protocol with 5 s at 95 °C and 20 s at 60 °C after an initial 5 min DNA melting step at 95 °C and finishing with a melting curve analysis starting at 60 °C and increasing with 1 °C per cycle up to 95 °C in a RotorGene Q thermocycler (Qiagen, Venlo, The Netherlands). Standard curves for absolute quantifications were generated by quantifying purified PCR products. DNA concentration was determined by NanoDrop 1000 (Peqlab, Erlangen, Germany) measurements in triplicate and diluted to 10^−3^ to 10^−9^ ng/µL. A 1 µL portion of standards was used in qPCR reference reactions and the copy number was calculated using the amplified sequence [44].

### 2.3. Variability of Symbiont Titer Assessed by Fluorescence in Situ Hybridization (FISH)

FISH experiments that were conducted over the years were compared to verify the variable symbiont titer via bacteriome structure and related symbiont numbers. All examples were derived from females from the JKI stock culture that were fixated overnight in 4% paraformaldehyde in PBS. After washing in PBS, individual specimens were immediately dehydrated and embedded in Technovit 8100 (Heraeus Kulzer, HAnau, Germany). Sagittal 10 µm sections were cut on a microtome (Microtome HM355S, Leica, Wetzlar, Germany) and mounted on diagnostic microscope slides.

Sections were covered with hybridization buffer containing 0.9 M NaCl, 0.02 M Tris/HCl, 0.01% SDS, 0.5 μM of each labelled oligonucleotide probe (OsurSym_16S labelled with Cy3 specifically staining the symbiont and Eub388 labelled with Cy5 staining a wider spectrum of bacteria [44], and 5 μg/mL of the general DNA stain DAPI. Hybridization was performed at 50 °C for 60 min and sections were subsequently washed twice with washing buffer consisting of 0.1 M NaCl, 0.02 M Tris/HCl, 5 mM EDTA, and 0.01% SDS including a 20 min incubation step at 50 °C in the first washing buffer round, followed by a washing step with distilled water. After drying, the sections were covered with Vectashield (Vector Laboratories, Curlingham, CA, USA) and a cover slip. Observation and image acquisition were carried out with an AxioImager Z2 (Zeiss, Jena, Germany), equipped with a SOLA light engine LED light source (Lumencor, Beaverton, OR, USA) and in one case with an Apotome.2 (Zeiss, Jena, Germany) under 200–400× magnification with the Z-stack option.

### 2.4. Symbiont Influence on Adult Mortality and Reproduction

The first dataset on the influence of symbionts on the onset of reproduction was available on experimental cultures JKI-1 from the collection of eggs for monitoring of the larval development, as freshly hatched females were used to collect eggs daily. Adult survival and lifetime reproductive output were quantified beside the onset of reproduction from the experimental cultures JKI-2. The technical and biological replicate cultures JKI-3, Mannheim, and Mainz were used to confirm findings on the onset of reproduction.

For all replicates, beetles were collected during their fifth larval instar from the different experimental cultures and reared individually in 24-well plates until adults emerged. Individuals were sexed using the presence of a spike in male individuals on the femur of the hind legs following Halstead [47] and males were marked with a yellow dot on their thorax. As females do not start oviposition in their first week regardless of symbiont infection (personal observations), they were kept in mixed cultures with males for the first week to mate and then transferred individually to 12-well plates. The bottom of these wells was covered with a dried slurry of ground oat flakes (Bio-Hafergold, Holo, Germany) and the upper third of the walls was coated with Fluon (Sigma-Aldrich, St. Louis, MI, USA) to prevent females from escaping. Females prefer to deposit eggs hidden into small grooves, often within oat flakes. As these are hard to observe, we provided artificial cardboard traps (black cardboard pieces of 3 × 12 mm folded in a Z shape) for egg deposition that were easy to observe under a stereo-microscope (Wild Heerbrugg, Wetzlar, Germany). As it is unknown whether females obtain enough sperm to fertilize eggs for their entire life from a single mating, they were granted access to males every four weeks by placing a marked male for three days into their well. All experimental setups were monitored three times per week until first oviposition occurred. The experiment with the JKI-2 populations was further observed for the first 120 days in the same intervals for eggs, and females were transferred to a new well every six days. From day 120 on, wells were monitored every week and females were also transferred every week to a new well. Eggs and hatched offspring remained in their initial well for the entire juvenile stage until adults emerged from pupae. Thus, the output of eggs, but also of offspring reaching their reproductive phase, was monitored for each single female individually. The experiment was started with a total of 84 symbiotic and 84 aposymbiotic females and 70 aposymbiotic and 62 symbiotic males, but all individuals that died within the first week of the experiment were removed and not considered for the reproduction analysis assuming that the early death occurred due to experimental handling (including sexing, marking, and transfers). Additionally, several individuals escaped during the 9-month duration of the experiment and thus had to be removed for lifetime reproduction results. Female reproductive output (number of laid eggs), reproductive success (number of adult offspring), and offspring survival as well as female and male survival curves were obtained from this experiment.

### 2.5. Statistical Analyses

All statistical analyses were performed with RStudio (Version 3.5.0, RStudio, Boston, MA, USA). Data distribution was assessed both visually and by Shapiro–Wilk tests. Consequently, symbiont titer between selected life stages, developmental times of animals, total number of deposited eggs, and total number of adult offspring between symbiotic and aposymbiotic animals were compared with Wilcoxon rank sum tests including correction for false discovery rates (FDRs) by repeated testing following the Benjamini–Hochberg procedure [48], implemented in the R package ‘stats’. Survival rates were analyzed by manually calculated χ^2^ tests. Survival of aposymbiotic vs. symbiotic females and males as well as onset of reproduction were analyzed with Mantel–Cox tests (R packages ‘survival’ and ‘coxme’). R packages ‘ggplot2′ and ‘rms’ were used to visualize data.

## 3. Results

### 3.1. Symbiont Titer during Development

**Figure 1:**
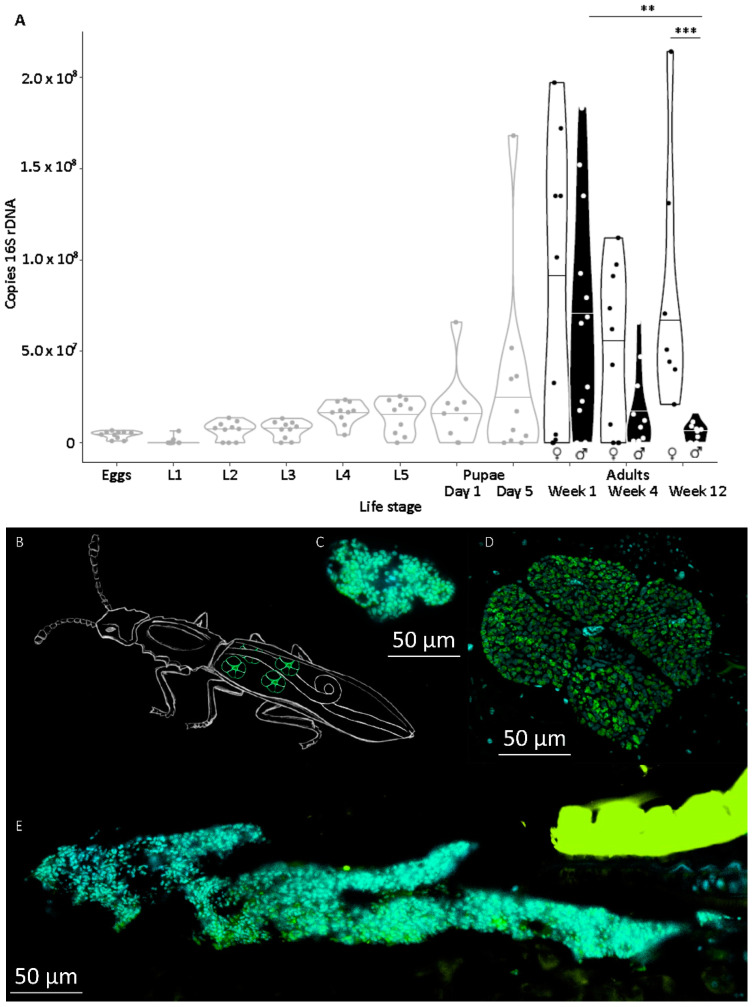
(**A**) Symbiont titer in different life stages of *O. surinamensis* from the Julius-Kühn-Institute/Federal Institute for Cultivated Plants (JKI) stock line. Symbiont titers were measured as 16S rDNA copies by PCR in single individuals. Juvenile life stages (eggs, larvae, and pupae; grey contours) contained mixed sexes, adults were separated by sex (black contours = females, black filling = males). Lines within ‘violin contours’ indicate median of respective life stage. For clarity, only significant differences of Wilcoxon rank sum test corrected for false discovery rate (FDR) following Benjamini–Hochberg are shown: * = *p* < 0.05, ** = *p* < 0.01, *** = *p* < 0.001. (**B**) Schematic representation (by Kathrin Hüffmeier) of the beetle body with the position and usual morphology of the four bacteriomes highlighted in green. (**C**–**E)** Examples of different-sized female bacteriomes (**C** smaller than usual, **D** usual, **E** unusually large and misshapen bacteriome) and according to symbiont titer visualized by fluorescence in situ hybridization in female beetles. Sagittal sections of female beetles with symbionts stained by a symbiont specific oligonucleotide probe labelled with Cy3 (green). DAPI was used to generally stain double-stranded DNA (cyan). Yellow-green structure in **E** is a piece of auto-fluorescent cuticle. Size bars equal 50 µm.

The symbiont titer showed a drastic decline from the initial median of 5.0 × 10^6^ copies of 16S rDNA of the Bacteroidetes symbionts in eggs, almost at the detection threshold in 1st instar larvae before increasing slowly to 1.8 × 10^7^ copies in 5th instar larvae. While the titer decreased slightly in pupae, both male and female adults showed a similar ~4-fold increase in bacteria within the first week after eclosion (7.8 × 10^7^ copies and 7.1 × 10^7^ copies, respectively, Figure 1A). In females, this titer was retained over the next months without significant changes (Wilcoxon rank sum tests in comparison to week 1; week 4: 4.9 × 10^7^ copies W = 65, p_BH corrected_ = 0.392; and week 12: 8.2 × 10^7^ copies W = 30, p_BH corrected_ = 0.770; Figure 1A), while the titer in males was significantly reduced by a factor of ten over time (Wilcoxon rank sum tests in comparison to week 1; male week 4: W = 93, p_BH corrected_ = 0.0699; and male week 12: W = 104, p_BH corrected_ = 0.00926, Figure 1; comparison male–female week 12: W = 70, p_BH corrected_ = 0.000720; Figure 1A). Surprisingly, adult beetles exhibited within the same age group relatively variable symbiont titers, indicated by qPCR results (Figure 1A), but also confirmed by fluorescence in situ hybridization of female beetles of variable age (Figure 1B–E). While D shows a bacteriome of usual structure and size, the bacteriome in C is unusually small and the one in Figure 1E is enlarged and misshapen. The calculated relative volume of the bacteriomes, assuming a similar extent in all three dimensions, is 1:8.8:15.4, reflecting more than one order of magnitude of difference, without taking, for example, symbiont density into account.

### 3.2. Symbiont Influence on Development

**Figure 2:**
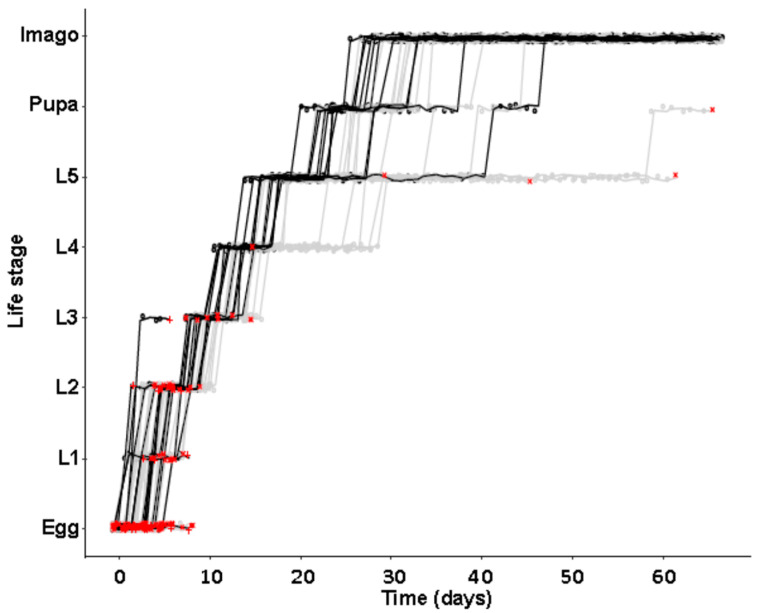
Cumulative developmental time until the end of different life stages of aposymbiotic (grey dots and lines) versus symbiotic (black dots and lines) *O. surinamensis* beetles from the JKI-1 lines. Red ‘+’ and ‘×’ signs indicate death of symbiotic and aposymbiotic individuals, respectively. Significant differences were observed for transitioning from larval instars 3 to 4 and instar 4 to 5 after Wilcoxon rank sum test corrected for FDR following Benjamini–Hochberg (* = *p* < 0.05, all other comparisons *p* > 0.05).

Aposymbiotic larvae showed a growing cumulative delay in development in the absence of symbionts, reaching significant differences of 1.5 and 2.5 days until instar 3 and 4 were completed (Wilcoxon rank sum tests; 3rd instar: W = 133, p_BH corrected_ = 0.0433; 4th instar W = 138, p_BH corrected_ = 0.0433). However, this delay did not persist in later life stages (*p* > 0.05; Figure 2), due to the most delayed individuals ultimately dying before finishing their development.

### 3.3. Symbiont Influence on Adult Life Span and Reproduction

**Figure 3:**
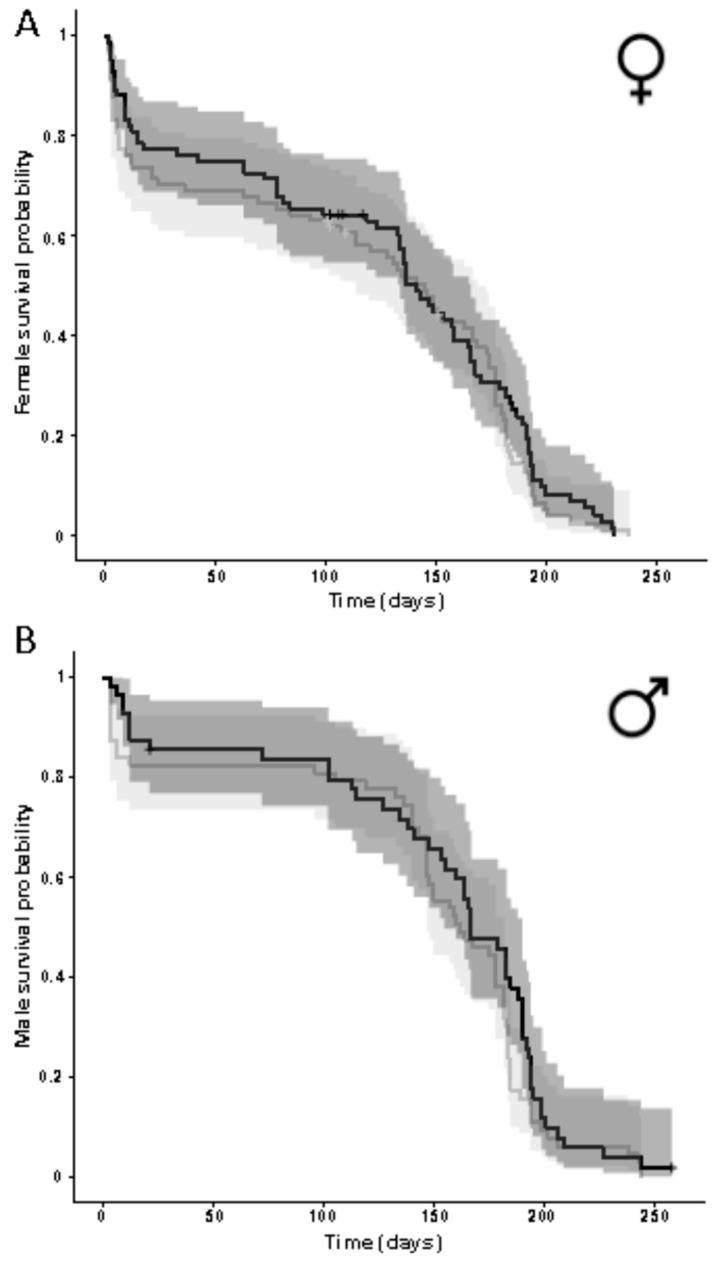
Survival of (**A**) female and (**B**) male adult *O. surinamensis* beetles with (black lines and dark grey shaded areas) and without (grey lines and light grey shaded areas) symbionts from the JKI-2 experimental lines. ‘+’ signs indicate censored individuals that were lost during the experiment. Shaded areas indicate 95% confidence intervals. No significant differences between symbiotic and aposymbiotic beetles were observed for neither females nor males (Cox regression, *p* > 0.05).

Symbiont presence influenced neither female nor male adult life time or mortality (Cox regression; females: N_apo_ = 84, N_sym_ = 84, coefficient_sym_ vs. _apo_ ± SE = −0.110 ± 0.0162, Wald z = −0.683, *p* = 0.495; males: N_apo_ = 63, N_sym_ = 56, coefficient_sym_ vs. _apo_ ± SE = 0.226 ± 0.191, Wald z = 1.19, *p* = 0.236; Figure 3).

**Figure 4:**
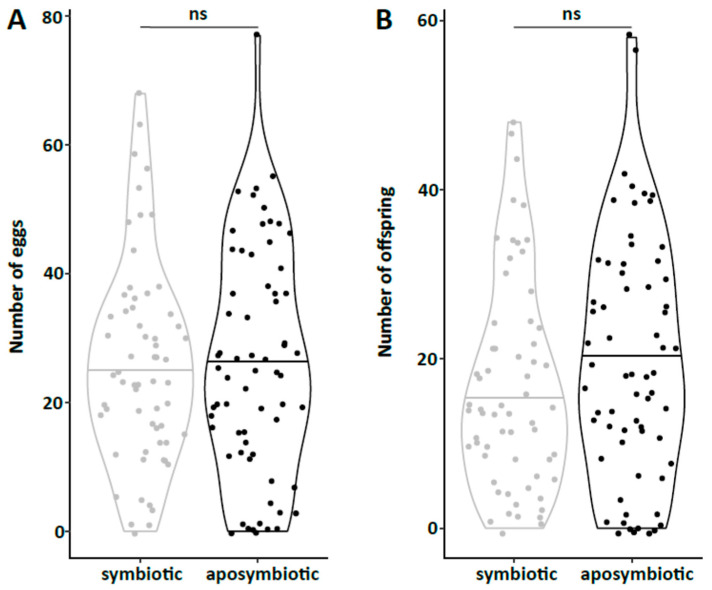
Influence of symbionts on reproductive traits of aposymbiotic (grey contours) and symbiotic (black contours) *O. surinamensis* females from the JKI-2 experimental lines. Shown is (**A**) the lifetime reproductive output (number of eggs) of a single female and (**B**) the number of offspring from a single female that reached the reproductive (adult) stage. Lines in contours indicate median of the respective sample group. No significant differences were observed using Wilcoxon rank sum test (ns = *p* > 0.05).

The lifetime reproduction of females from the JKI-2 experimental cultures did not differ based on symbiont presence, neither regarding the amount of laid eggs (Wilcoxon rank sum test, W = 2074, p_BH corrected_ = 0.896, Figure 4A), nor the number of offspring reaching adulthood and thus representing potentially reproductive next generation (Wilcoxon rank sum test, W = 1801.5, p_BH corrected_ = 0.285, Figure 4B), even though aposymbiotic larvae exhibited a higher mortality over their entire development (apo 32.1% (N = 1611), sym 38.3% (N = 1699); χ^2^ homogeneity test: χ^2^ = 14.0, d.f. = 1, *p* < 0.001, offspring of experiment JKI-2).

**Figure 5:**
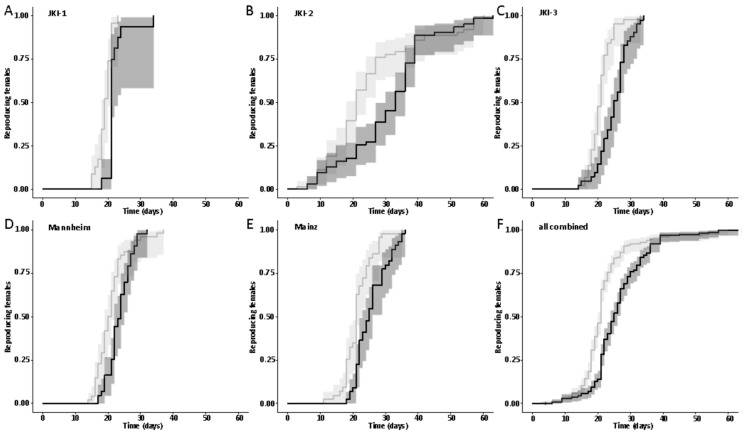
Influence of symbionts on the onset of reproduction in different *O. surinamensis* lines ((**A**–**C**) JKI, (**D**) Mannheim, (**E**) Mainz). Symbionts in the JKI line were eliminated three times independently, resulting in lines JKI-1, JKI-2, and JKI-3. Experiments with these lines were conducted at different times, in different incubators; experiments with lines Mannheim and Mainz were conducted at the same time as JKI-3. (**F**) Overall, aposymbiotic females (grey lines and light grey, 95% confidence intervals) started to reproduce significantly earlier than symbiotic females (black lines and dark grey, 95% confidence intervals; Cox regression: *p* < 0.05). The same result was observed in all single experiments (Cox regression: *p* < 0.05) except JKI-2 ((**B**) *p* = 0.306), where the majority of aposymbiotic females started earlier than symbiotic ones, but symbiotic ones started to ‘catch up’ towards the end of the experiment.

**Figure 6:**
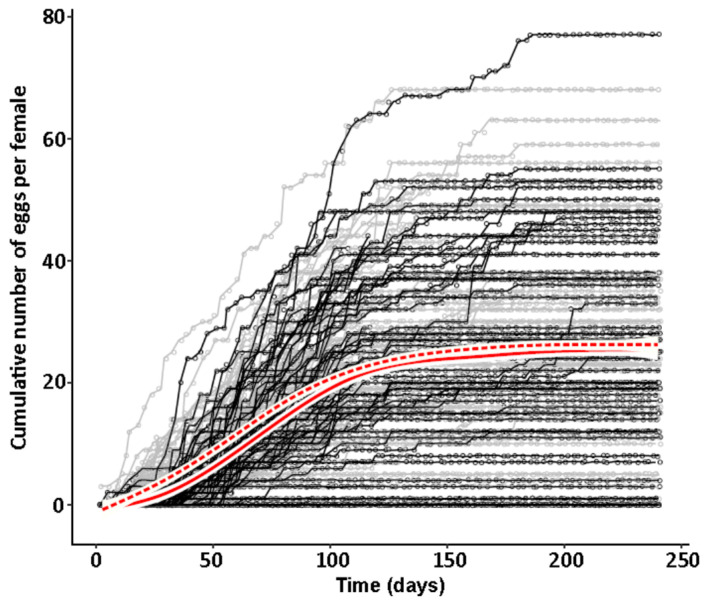
Time course of reproductive output (cumulative number of laid eggs) of single symbiotic (black circles and lines) versus aposymbiotic (grey circles and lines) *O. surinamensis* females from the JKI-2 experimental lines and average of both populations with 95% confidence intervals (symbiotic = continuous red line and white area, aposymbiotic = dashed red line and white area) reveal a window of approximately 90 days in which the reproductive output of symbiotic females lags behind that of aposymbiotic females by approximately 10 days.

However, while lifetime reproductive output did not differ, females started overall significantly earlier with oviposition in the absence of symbionts than in their presence (global test with culture strains/replicates as random variable - Cox mixed effects regression: N_apo_ = 219, N_sym_ = 206, coefficient_sym_ vs. _apo_ ± SE = −0.601 ± 0.0992, Wald z = −6.06, *p* = 1.4 × 10^−9^; Figure 5). Single cultures and replicates differed in the magnitude and exact course of the onset of reproduction over the entire population. In most, the first single aposymbiotic females started to reproduce before symbiotic ones, and the difference in reproducing females was constant over the entire experiment, whereas in some the first aposymbiotic and symbiotic females started at the same time, but more aposymbiotic females started to reproduce more quickly in the following days, and in some symbiotic females caught up towards the end of the experiment (Cox regressions for single strains/replicates: JKI-1, N_apo_ = 23, N_sym_ = 19, coefficient_sym_ vs. _apo_ ± SE = −1.13 ± 0.351, Wald z = −3.22, *p* = 0.0013; JKI-2 N_apo_ = 63, N_sym_ = 62, coefficient_sym_ vs. _apo_ ± SE = −0.184 ± 0.178, Wald z = −1.02, *p* = 0.306; JKI-3 N_apo_ = 42, N_sym_ = 41, coefficient_sym_ vs. _apo_ ± SE = −1.03 ± 0.232, Wald z = −4.45, *p* = 8.42 × 10^−6^; Mannheim N_apo_ = 43, N_sym_ = 44, coefficient_sym_ vs. _apo_ ± SE = −0.454 ± 0.216, Wald z = −2.11, *p* = 0.0351; Mainz N_apo_ = 48, N_sym_ = 43, coefficient_sym_ vs. _apo_ ± SE = −0.823 ± 0.223, Wald z = −3.64, *p* = 0.000273; Figure 5). The earlier onset of reproduction of aposymbiotic females caused a window of approximately 90 days in which aposymbiotic females laid on average 40% more eggs than symbiotic ones (JKI-2, Figure 6).

## 4. Discussion

The sawtoothed grain beetle *O. surinamensis* engages in an ancient symbiosis with cuticle supplementing Bacteroidetes bacteria [43,44]. Symbiotic beetles exhibited a context-dependent higher desiccation tolerance, especially a lower mortality during larval development [44]. In this study, we set out to test whether the symbiosis also caused costs for the host. Therefore, we monitored larval development as well as adult life history traits, including mortality and reproduction. In addition, we quantified symbiont growth (titers per individual) during development and adult life. We specifically chose low desiccation stress (high humidity) to address the question of whether symbiont infection could, under natural conditions, be disadvantageous and selected against.

Symbiont titers increased mildly during larval development and metamorphosis, but drastically between the end of metamorphosis and in one-week-old adults. This level of symbiont titers was maintained in females throughout the tested period but declined again in males. We previously observed an important symbiont contribution to larval survival, most likely through supported cuticle synthesis. However, the need for such support is probably highest for *O. surinamensis*, but holometabolous insects in general, during metamorphosis, when the entire insect body is reshaped, and as young adults, when the initially soft and weak cuticle is strengthened via sclerotization and melanization [22,43,49]. This would correlate with a control, at least partial, of the symbiont contribution via symbiont titer, but also means that the highest investment of the host is required during metamorphosis, when nutrients must be also invested into the reshaping of its own body, and no novel nutrients can be acquired until imagines hatch and are fully hardened. Thus, potential cost can be assumed to manifest after this symbiont growth, until the host can compensate for this additional investment. Considering that the major physiological contribution of the symbiont occurs during larval development and metamorphosis for cuticle synthesis and the adult body will not undergo further development, maintaining the obsolete symbionts would be a waste of nutrients. Accordingly, males seem to recycle the symbiont, which was also observed in the grain weevil *Sitophilus oryzae* [22]. Females, however, need to maintain symbionts during their reproductive phase to transmit them to their offspring, giving reasons to expect costs especially in female life history traits, but less in the males’ traits.

Our observations of different life history traits lie well within previous descriptions. Development was described to take 21–51 days [42,50], adult lifespan up to two, seven, or up to six to ten months [51,52,53], for single individuals up to three years [51]. Females were reported to start oviposition after approximately one week [50] with up to ten eggs per day, but only a highly reproductive timespan of two to three weeks [50] that drastically levels off afterwards with occasional oviposition up to six months [54]. Lifetime reproduction of females varies accordingly between studies from 45–300 eggs per female [50,51,52,53]. While we observed a similar benefit of symbiont infection on larval mortality as previously reported [44], there was no difference in development time until adulthood, despite a gradual increase in symbiont numbers during larval development, as well as a stronger increase during metamorphosis. Neither was adult mortality nor lifespan influenced by the symbiont presence. However, while collecting eggs to monitor the larval development, we observed that symbiotic females started to oviposit significantly later than aposymbiotic beetles. As this was a small dataset with a different focus, we replicated this experiment with in total three biological replicates of the same beetle lab strain (eliminating symbionts in three separate batches) as well as two further wild-caught strains and observed overall that most symbiotic females started to oviposit several days later than aposymbiotic ones. For one population, we also compared lifetime reproduction of females. The later onset of reproduction in symbiotic beetles translated here in a delay of population growth that was maintained until both populations reached their maximum reproductive output, which did not differ between symbiotic and aposymbiotic females, neither when counting eggs, nor adult offspring, despite a higher mortality of aposymbiotic larvae.

Microbial symbionts of multicellular organisms provide diverse benefits but also entail certain costs [19,20]. For this study, we demonstrated that the nutritional symbionts of *O. surinamensis* entail effectively a time cost, delaying reproduction of beetles by several days to weeks. As the nutritional endosymbiont genomes usually encode only a minimal metabolism, they require basic nutrients for the synthesis of the cellular components from their host. Thus, the time delay represents the investment of nutrients into symbiont growth, which are later lacking and have to be acquired by the mature females before they can start to reproduce. Whether the time delay is costly for the host is highly context dependent. As the number of offspring did not differ between symbiotic and aposymbiotic females, the delayed onset of reproduction would not be costly in the case of individuals living in absolute isolation. However, multiple scenarios are conceivable in which it is advantageous to reproduce earlier in life. One important factor is intraspecific competition that can arise when beetles colonize novel, especially short-lived resource deposits, but also due to cannibalism of larvae, especially in high density [55]. In both scenarios, individuals would have the benefit of being older due to an earlier birth; larvae, because the older and larger larvae are stronger when preying on conspecifics; and adults that finish metamorphosis earlier would in turn be able to reproduce earlier. Thus, the disadvantage of harboring symbionts can also become costly in this scenario in the case where the symbiotic benefit of an enhanced cuticle decreases, e.g., at low desiccation stress. On the contrary, natural mortality, as observed especially in the early and late adult life in this study, but even higher adult mortality due to the presence of predators, parasitoids, or pathogens under natural situations, would represent selective pressure on early reproduction. This assumes that mortality does not differ between symbiotic and aposymbiotic beetles. On the other hand, the symbiont-enhanced cuticle synthesis offers superior protection, probably only from a certain strength of the cuticle and thus age of the maturing adult beetle, where selection may favor high symbiotic titers. However, as beetles likely experience a continuum of selective pressures, either selection of the individual insects based on a genetically fixed state of symbiont titers or plastic regulation of symbiont titers may represent a natural feedback mechanism on the symbiotic association of *O. surinamensis*.

Metabolic and consequent fitness costs of harboring symbionts have been repeatedly described in defensive symbioses, where symbiotic insects actually suffer diverse fitness costs in absence of the natural enemies against which the symbionts usually protect [15,37,56,57]. Sinotte et al. [58] also report on immunological costs due to an increased susceptibility of *Camponotus floridanus* against the entomopathogen *Metarhizium brunneum* in the presence of their *Blochmannia* endosymbionts, while *Blochmannia* otherwise supports cuticle synthesis, growth, and development. To our knowledge, no experimental demonstration of such costs yet exists for nutritional symbionts. In addition, the comparison of symbiotic and aposymbiotic hosts that is usually used in experimental studies to quantify costs is for most nutritional symbioses an artificial construct—in contrast to many defensive ones. However, aposymbiotic subpopulations have been reported in *O. surinamensis* due to the symbionts’ sensitivity to already mild increases in substrate temperature of above 30 °C, which can easily be caused by concomitant fungal infestations in grain storages [23,24]. Furthermore, we observed in this study a high variation in symbiont titer between individuals, e.g., a 5 × 10^5^-fold difference in one-week-old females, which constitutes an obvious trait for natural selection as cuticle supplementation was related to symbiont titer in the palm weevil *Pachyrhynchus infernalis* and its *Nardonella* symbiont [59]. Thus, depending on environmental conditions, female beetles with high symbiont benefits (equaling high titer) or low costs (low titer) can easily be selected through this standing variation in symbiont titer. Most natural scenarios will of course present conditions somewhere in between both extremes, presenting fluctuating selective pressure on the evolution of the entire beetle–bacteria association that could lead to variable infection frequencies, as described in several defensive symbioses [15,16,17].

## 5. Conclusions

We provide the first quantitative report on several life history parameters that are specific to the symbiosis of *O. surinamensis*. On the symbiont side, we find that low titers continuously increase during larval development until the onset of metamorphosis. Towards the end of metamorphosis, titers increase again until reaching their maximum in young adults with no difference between females and males. The high titers are retained in females, whereas they decline again with age in males. Notably, the symbiont titer is highly variable within each tested life stage, but mostly in adults. On the host side, there is no influence of symbiont presence on larval developmental time, adult mortality, or lifespan, but symbiont presence seems to incur costs to females, leading to a delayed onset of reproduction of one to two weeks.

These metabolic costs that can easily translate into fitness costs under various scenarios present, together with previously reported influence of symbiont presence on cuticle formation and thereby desiccation susceptibility of larvae [44], context-dependent benefits or costs of harboring an intracellular symbiont. Furthermore, the standing variation of symbiont titer between individuals represents a selective trait acting in favor of symbiont maintenance or against it. In combination with the experimental amenability, namely straight-forward mass rearing, transgenerational manipulation of the symbiont titer or presence, and a relatively short life cycle, the symbiosis of *O. surinamensis* represents a system that is well suited to study eco-evolutionary consequences or dynamics of symbiotic traits under variable selection pressures.
